# Cyclical variation in the national incidence of childhood type 1 diabetes in Australia (2000 - 2011)

**DOI:** 10.1186/1687-9856-2015-S1-P14

**Published:** 2015-04-28

**Authors:** Aveni Haynes, Max K Bulsara, Carol Bower, Timothy W Jones, Elizabeth A Davis

**Affiliations:** 1 Department of Endocrinology & Diabetes, Princess Margaret Hospital, Perth, WA, Australia; 2 Telethon Kids Institute, The University of Western Australia, Perth, WA, Australia; 3 Institute of Health and Rehabilitation Research, University of Notre Dame, Fremantle, WA, Australia; 4 School of Paediatrics and Child Health, The University of Western Australia, Perth, WA, Australia

## 

To determine the incidence and incidence rate trends of type 1 diabetes in children aged 0-14 years Australia-wide using all available data from 2000 to 2011, and to examine the temporal trends for non-linear, cyclical variation.

Cases were identified from the National Diabetes Register (NDR) which was established in 1999 and contains data on patients with insulin-treated diabetes Australia-wide. For 0-14 year olds with type 1 diabetes the NDR is estimated to be 97.7% complete [1]. Population data published by the Australian Bureau of Statistics were used as the denominator data. Annual age-standardised as well as gender and age specific incidence rates were calculated. Poisson regression was used to analyse the incidence by calendar year, gender, and age at diagnosis. To analyse the incidence for non-linear variation, sine and cosine functions were applied to Poisson regression models for 3-, 4-, 5-, 6-, and 7-year cycles and the Akaike Information Criterion (AIC) used to assess goodness-of-fit [2].

Between 2000 and 2011, 11,575 (6,049 M, 5,526 F) cases of childhood type 1 diabetes were identified from the NDR. The mean incidence of childhood type 1 diabetes in Australia over this time period was was 23.6 (95%CI:23.2 – 24.0) per 100,000 person years. The mean incidence was 4.9% (95%CI:1.1% - 8.8%) higher in boys compared to girls. Compared to 0-4 year olds, the mean incidence was 65% higher in 5-9 year olds, and 208% higher in 10-14 year olds. No significant linear increase in the incidence rate trend was observed overall (Incidence rate ratio 1.003 (95%CI:0.997 – 1.008), or by gender. An average annual increase in incidence was only observed in the 10-14 year old age group (1.2% per year (95%CI:0.4% - 2.1%)). When analysed for non-linear variation in the temporal trends, a 5-year cyclical variation of 6% was observed in the overall incidence rate trend. A 5-year cyclical variation in incidence was observed in both genders and all age groups.

From 2000 to 2011, no linear increase in the annual overall incidence of childhood Type 1 diabetes was observed in Australia. Of interest, a sinusoidal pattern in the incidence rate trend was observed, with a 5-year cyclical pattern. The cyclical pattern of incidence rate trends observed Australia-wide corroborates findings reported in Western Australia [3] and provides further evidence supporting a key role of environmental factors in the aetiology or clinical onset of childhood type 1 diabetes – further investigation is required.
